# The prevalence and correlates of unintended pregnancy among female sex workers in South China: a cross-sectional study

**DOI:** 10.1186/s12978-024-01853-7

**Published:** 2024-07-24

**Authors:** Peng Liang, Peizhen Zhao, Yijia Shi, Shujie Huang, Cheng Wang

**Affiliations:** 1 Dermatology Hospital, Southern Medical University, Guangzhou, 510095 China; 2 School of Public Health, Southern Medical University, Guangzhou, 510515 China; 3 Southern Medical University Institute for Global Health, Guangzhou, 510095 China; 4 Johns Hopkins Bloomberg School of Public Health, Baltimore, USA

**Keywords:** Unintended pregnancy, Induced abortion, Female sex workers, China

## Abstract

**Background:**

Female sex workers (FSW) are particularly vulnerable to unintended pregnancy. Research examining the experience of unintended pregnancy due to commercial sex among Chinese FSW, however, is limited. This study aimed to examine the prevalence and correlates of unintended pregnancy due to commercial sex among FSW in China.

**Methods:**

In 2021, a cross-sectional study was conducted among 1257 FSW in five cities from Guangdong provinces in South China. Data were collected on social-demographic characteristics, sexual behaviors, experience of unintended pregnancy due to commercial sex and its pregnancy outcome, as well as experience of abortion in lifetime. Multivariable logistic regression analysis was employed to identify factors associated with unintended pregnancy.

**Results:**

Among the 1257 FSW, 19.3% reported having at least one unintended pregnancy due to commercial sex. Of those, 96.7% chose to terminate the pregnancy through induced abortion, and 40.5% reported undergoing multiple induced abortions in their lifetime. Multivariable logistic regression indicated that FSW working in current location over one year (adjusted Odds Ratio (aOR): 2.82, 95% CI 1.71–4.64) and having more than seven clients in the past week (aOR: 4.53, 95% CI 2.74–7.51) were more likely to have had unintended pregnancy due to commercial sex. Working in high tier (aOR: 0.21, 95% CI 0.14–0.30) and consistent condom use with clients in the past month (aOR: 0.16, 95% CI 0.10–0.23) were associated with a lower proportion of FSW having ever had unintended pregnancy.

**Conclusions:**

Unintended pregnancy are prevalent among FSW in South China. Interventions aimed at reducing the prevalence of unintended pregnancy and enhancing post-abortion care could be necessary among Chinese FSW.

## Abstract (plain language summary)

Female sex workers (FSW) are particularly vulnerable to unintended pregnancies. Research on the experience of unintended pregnancy resulting from commercial sex among Chinese FSW is, however, limited.

To address this knowledge gap, we investigated the prevalence and correlates of unintended pregnancies due to commercial sex among FSW in China. We conducted a cross-sectional study among 1257 FSW in five cities across Guangdong Province in South China in 2021. Multivariable logistic regression analysis was used to identify factors associated with unintended pregnancy.

Among 1257 FSW, 19.3% reported having experienced at least one unintended pregnancy due to commercial sex. The factors significantly associated with unintended pregnancy in the multivariate analysis included participants working in high tier, working in current location over one year, using condom with clients inconsistently in the past month, and having more clients in the past week.

The findings from this study could provide valuable insights for the development of policies aimed at reducing unintended pregnancies, improving abortion care, and enhancing family planning programs targeted at FSW.

## Introduction

Female sex workers (FSW) are vulnerable to adverse sexual and reproductive health outcomes due to frequent sexual encounters with multiple sex partners, and challenges in negotiating condom use with clients [1]. This heightened vulnerability exposes them to an increased risk of contracting human immunodeficiency virus (HIV) and other sexually transmitted infections (STIs) and experiencing unintended pregnancy. Unintended pregnancy was defined as any pregnancy deemed by the woman to be unintended or undesired at the time of conception, based on evaluations made prior to pregnancy [2]. The prevalence of unintended pregnancy among FSW was found to be high in Zambia (61.6% in 2016) [3], Cameroon (57.6% in 2016) [4], and Ethiopia (28.6% in 2014) [5]. In China, the estimated prevalence of unintended pregnancy among FSW was 46.5% in 2012 [6] and 11.3% in 2020 [7].

FSW often opted for induced abortion as a means for pregnancy termination, driven by factors such as the fear of client attrition, intimate partner violence, economic duress, and societal opprobrium [8]. A study conducted in Benin suggest that approximately 28% of FSW have encountered unintended pregnancies, with substantial approximately 60% of these pregnancies ended in abortion [9]. Abortion, particularly when conducted under unsafe conditions, can have severe consequences for women, including maternal mortality and various health complications. A global systematic review has indicated that abortion is a leading cause of death among FSW, accounting for approximately 65% of all maternal deaths and 35% of all deaths [10].

Studies and initiatives targeting FSW in resource-limited settings have predominantly concentrated on their susceptibility to HIV and STIs, often overlook this challenging aspect of the prevention of unintended pregnancy in FSW [11]. Additionally, discrimination from healthcare providers, coupled with FSW’s low health-seeking motivation due to social marginalization, may further exacerbate the barriers to receive substandard healthcare [12, 13]. Hence, interventions to mitigate unintended pregnancy are warranted, which should be designed based on a comprehensive understanding of the factors influencing unintended pregnancies from the perspective of FSW. However, research to date focusing on unintended pregnancy among Chinese FSW is rare and limited to report the prevalence rates without exploring the underlying associated factors [6, 7]. Hence, the purpose of this study was to examine experience of unintended pregnancy due to commercial sex and its associated factors among FSW in South China.

## Methods

### Study site

This cross-sectional study was conducted in Guangdong province in Southern China between April and August 2021. Guangdong Province was chosen for this study based on the high burden of STIs and abortion. Guangdong has been among the top three in China in terms of reporting new STIs cases over the past decade [14–16]. In addition, the number of induced abortions in Guangdong province reached 1.2 million in 2018, which ranked first in the number of induced abortion cases in China [17].

### Study participants

This study was implemented in five cities (Yunfu, Jiangmen, Rongcheng, Puning, and Yingde). These cities were selected based on the size of FSW and the high burden of STIs. Each city has a professional local outreach team that provides HIV/STIs and reproductive health-related services, including HIV/STIs testing and prevention, condom promotion, sex education, and contraceptive counseling. Eligible participants included females assigned at birth, 18 years of age or older, who had traded sex for money or goods in the past year, and voluntarily provided informed consent.

### Sample size

The primary outcome of this study was the prevalence of unintended pregnancy due to commercial sex. The prevalence of unintended pregnancy among FSW assumed to be 11.3% based on a previous study [7]. We utilized the Two-sided Confidence Intervals (CI) for One Proportion method to estimate a minimum sample size of 963 for this study, ensuring a two-sided 95% CI with a width of 0.020. According to the number of FSW at each city, the total number of study participants at each city ranged from 200 to 250.

### Sampling design and procedure

Prior to this study, local outreach team members created a distribution map of sex work venues through a preliminary recognition of geographic area, type of venue, and size estimation. Sex work venues were divided into two primary tiers: high tier venues offering both life and sexual services (such as saunas, nightclubs, karaoke bars, and hotels), and middle and low tier which only provide sexual services (such as foot bathing shops, hair salons or barber shops, roadside shops, and streets or public outdoor places). Subsequently, venues in each city were randomly chosen through proportional sampling. The local outreach team members then personally visited these designated tiers, extending invitations to FSW to engage in the survey. The FSW count per city varied from 200 to 250.

### Data collection

A paper-based questionnaire was used for data collection in this study. Items in the questionnaire were designed and formulated based on literature reviews and discussions by STIs and sexual and reproductive health experts. Before the formal study, a pilot survey was conducted by our team to validate the reliability and viability of questionnaires. Meanwhile, the feasibility of the main survey processes and the capacity of the local outreach team were also tested to ensure smooth recruitment and data collection. The data from the pilot survey were excluded in the final analysis.

All eligible participants completed the survey questionnaire anonymously in a private room after signing the written informed consent. During the study, team members provided pre- and post-counseling regarding STIs and reproductive health in case participants had any inquiries. After completing the questionnaire survey, the outreach members collected specimens from all participants. All urine and blood samples were transported to the Guangdong Provincial Center for Skin Disease and STD Control for analysis. A provider-collected urine sample was used to test for N. gonorrhoeae/C. trachomatis using nucleic acid amplification testing (NAAT), and a blood sample was used for syphilis testing through a rapid plasma regain (RPR) test. If RPR is positive, the Treponema pallidum particle agglutination (TPPA) test will be performed for syphilis confirmation. Laboratory results were typically available within two weeks following specimen receipt. Participants who had a positive test result were directed to undergo confirmatory laboratory testing and a clinical examination at designated clinics or hospitals to ensure accurate diagnosis and appropriate treatment.

### Measures

#### Sociodemographic and sexual behaviors characteristics

Sociodemographic information included age, marital status, education level, monthly income, workplace, length of time working in the current location, and place of domicile. Sexual behaviors variables included the mean number of clients served in the past week, whether consistent condom use with clients in the past month, whether participants sought clients for commercial sex via online platforms, the main contraceptive methods, and STIs testing results. Consistent condom use was defined as always using a condom during commercial sex. The prevalence of combined STIs was defined as having a positive testing for at least one of the STIs (syphilis, chlamydia or gonorrhea).

#### Unintended pregnancy and induced abortion

Self-report measures were used to record the prevalence of unintended pregnancy and induced abortion. The occurrence of unintended pregnancy resulting from commercial sex was classified as a binary variable, with participants responding to the query, “Have you experienced unintended pregnancy due to commercial sex?”. Subsequently, participants were asked about their primary pregnancy outcomes in case of unintended pregnancy occurred due to sex work, encompassing induced pregnancy, spontaneous labor, spontaneous abortion, and exfetation. Additionally, participants also self-reported the total number of induced abortions in their lifetime. Multiple abortions were defined as having at least two abortions in their lifetime.

### Statistical analysis

We used descriptive statistics on sociodemographic information, sexual behaviors, the prevalence of STIs, the history of unintended pregnancy, primary pregnancy outcome, and lifetime induced abortion, with x̄ ± SD for continuous variables and the number and percentage for categorical variables. Chi-square tests were performed to compare differences in categorical variables between groups.

Univariable and multivariable logistic regression was carried out to investigate the factors associated with unintended pregnancy resulting from commercial sex. Covariates including age, education level, monthly income, workplace, and length of time working in the current location were included in the multivariable models on the basis of a review of the literature [18, 19]. Furthermore, a sub-analysis was conducted to examine the occurrence of multiple abortions in the lifetime among participants who had previously experienced unintended pregnancy due to commercial sex. We reported odds ratios (OR), adjusted odds ratios (aOR), 95% confidence intervals (CI), and P values. P-values ≤ 0.05 were considered statistically significant. All analyses were performed using IBM SPSS Statistics version 26.

## Results

### Sociodemographic characteristics

A total of 1337 women met the inclusion criteria for the study. Among them, 81 individuals declined to participate. Ultimately, 1257 FSW completed the survey, indicating a response rate of 95% (1257/1337). The mean age of the participants was 31.61 ± 8.85 years, with ages ranging from 18 to 68 years. Nearly one-third of the participants were 36 years old or older. Most participants were unmarried, had a middle school degree, had a monthly income between 3000¥ and 5000¥, and being from high tier venues (Table 1).

**Table 1:** Demographics and sexual behaviors of FSW in Guangdong Province, China, 2021 (*N* = 1257)

Characteristics	Total n (%)	Unintended pregnancy due to commercial sex		*P*
		Yes n (%)	No n (%)	
Demographics	1257	242 (19.3)	1015 (80.7)	
Age				< 0.001*
Mean ± SD	31.61** ± **8.85	35.29 ± 9.26	30.73** ± **8.52	
18–22	215 (17.1)	19 (7.8)	196 (19.3)	
23–28	309 (24.6)	51 (21.1)	258 (25.4)	
29–35	332 (26.4)	53 (21.9)	279 (27.5)	
≥ 36	401 (31.9)	119 (49.2)	282 (27.8)	
Marital status				< 0.001*
Unmarried	508 (40.4)	71 (29.3)	437 (43.1)	
Married	469 (37.3)	83 (34.3)	386 (38.0)	
Divorced	280 (22.3)	88 (36.4)	192 (18.9)	
Education level				< 0.001*
Illiterate or elementary	254 (20.2)	87( 36.0)	167 (16.5)	
Middle school	721 (57.4)	115 (47.5)	606 (59.70)	
High school and above	282 (22.4)	40 (16.5)	242 (23.8)	
Monthly income (￥)				< 0.001*
< 3000	129 (10.3)	24 (9.9)	105 (10.4)	
3000–5000	538 (42.8)	159 (65.7)	379 (37.3)	
5000–8000	355 (28.2)	44 (18.2)	311 (30.6)	
> 8000	235 (18.7)	15 (6.2)	220 (21.7)	
Workplace				< 0.001*
Middle and low tier	579 (46.1)	194 (80.2)	385 (37.9)	
High tier	678 (53.9)	48 (19.8)	630 (62.1)	
Length of time working in current location				< 0.001*
< 6 months	292 (23.2)	22 (9.1)	270 (26.6)	
6–12 months	302 (24.0)	70 (28.9)	232 (22.9)	
Over 1 year	663 (52.8)	150(62.0)	513 (50.5)	
Place of domicile				0.171
Local city	103 (8.2)	14 (5.8)	89 (8.8)	
Other cities in this province	238 (18.9)	53 (21.9)	185 (18.2)	
Other provinces	916 (72.9)	175 (72.3)	741 (73.00)	
Have ever occurred a gynecological disease				< 0.001*
No	736 (58.6)	171 (70.7)	565 (55.7)	
Yes	521 (41.4)	71 (29.3)	450 (44.3)	
Have ever received any local health services regarding STIs				< 0.001*
No	262 (20.8)	69 (28.5)	193 (19.0)	
Yes	995 (79.2)	173 (71.5)	822 (81.0)	
Sexual behavior				
Number of clients (past week)				
Mean ± SD	7.73 ± 5.14	9.38 ± 4.64	7.34** ± **5.17	< 0.001*
≤ 3	278 (22.1)	24 (9.9)	254 (25.0)	
4–6	343 (27.3)	51 (21.1)	292 (28.8)	
≥ 7	636 (50.6)	167 (69.0)	469 (46.2)	
Consistent condom use with client (past month)				< 0.001*
No	568 (45.2)	203 (83.9)	365 (36.0)	
Yes	689 (54.8)	39 (16.1)	650 (64.0)	
Have ever looked for clients through online software?				< 0.001*
No	729 (58.0)	57 (23.6)	672 (66.2)	
Yes	528 (42.0)	185 (76.4)	343 (33.8)	
Currently the main approach of contraception				0.007*
Condom	1099 (87.4)	199 (82.2)	900 (88.7)	
Other contraceptive methods†	158 (12.6)	43 (17.8)	115 (11.3)	
Previous chlamydia testing				< 0.001*
No	919 (73.1)	95 (39.3)	824 (81.2)	
Yes	338 (26.9)	147 (60.7)	191 (18.8)	
Previous gonorrhea testing				< 0.001*
No	943 (75.0)	100 (41.3)	843 (83.0)	
Yes	314 (25.0)	142 (58.7)	172 (17.0)	
Syphilis testing				0.099
Negative	1236 (98.3)	235 (97.1)	1001 (98.6)	
Positive	21 (1.7)	7 (2.9)	14 (1.4)	
Chlamydia testing				0.664
Negative	1182 (94.0)	229 (94.6)	953 (93.9)	
Positive	75 (6.0)	13 (5.4)	62(6.1)	
Gonorrhea testing	0.028*			0.028 *
Negative	1183 (94.1)	235 (97.1)	948 (93.4)	
Positive	74 (5.9)	7 (2.9)	67 (6.6)	
Combined STIs testing	0.233			0.233
Negative	1104 (87.8)	218 (90.1)	886 (87.3)	
Positive	153(12.2)	24 (9.9)	129 (12.7)	

^*^P < 0.05

^†^such as oral contraceptive or long-acting contraceptive needle/ring

### Sexual behaviors

Of the 1257 participants, the average number of clients served in the past week was 7.73 ± 5.14. Half of participants consistently used condom when engaging in sex with clients in the past months. Almost two-fifths of participants reported ever using the online software to seek clients. Most participants reported that condom use was the main contraceptive method to protect themselves from unintended pregnancy (Table 1).

### The prevalence of STIs

The prevalence of syphilis, chlamydia and gonorrhea infections among FSW were 1.7%, 6.0%, and 5.9%. The prevalence of combined STIs was 12.2% (Table 1).

### Prevalence of unintended pregnancy and induced abortion

The proportion of participants with ever experiencing at least one unintended pregnancy due to commercial sex was 19.3%. Of the 242 participants, the majority of participants chose induced abortion as a measure to terminate unintended pregnancy. In addition, among those who experienced unintended pregnancy, about four-fifths of participants self-reported that they had experienced induced abortion in their lifetime, while 40.5% of participants had multiple induced abortion. The proportion of having an abortion once in their lifetime is the highest, followed by two abortions (Table 2).

**Table 2:** Overview of lifetime abortion among FSW who had ever unintended pregnancy due to commercial sex in Guangdong Province, China, 2021 (*N* = 242)

Variable	*Total (n)*	*%*
Pregnancy outcome	242	
Spontaneous labor		
No	240	99.2
Yes	2	0.8
Spontaneous abortion		
No	235	97.1
Yes	7	2.9
Induced abortion		
No	8	3.3
Yes	234	96.7
Exfetation		
No	240	99.2
Yes	2	0.8
Lifetime abortion		
Number of abortions		
0	27	11.2
1	117	48.3
2–3	82	33.9
4–5	16	6.6
More than one abortion		
No	144	59.5
Yes	98	40.5

### Factors correlated with unintended pregnancy

In the multivariable logistic analysis, adjusting for age, workplace, duration of work in the local area, education level and monthly income, participants who had more than seven clients in the past week (aOR = 4.53, 95% CI 2.74–7.51), looked for clients through online software (aOR = 4.96, 95% CI 3.40–7.23), previous tested for chlamydia (aOR = 3.71, 95% CI 2.67–5.18) and gonorrhea (aOR = 3.88, 95% CI 2.77–5.43) and used oral or long-acting contraception (aOR = 1.77, 95% CI 1.16–2.70) were more likely to have unintended pregnancy due to commercial sex. Conversely, working in high tier (aOR = 0.21, 95% CI 0.14–0.30) and consistent condom use with clients (aOR = 0.16, 95% CI 0.10–0.23) were associated with a lower proportion of FSW having ever had unintended pregnancy (Table 3).

**Table 3:** Factors associated with unintended pregnancy among FSW in Guangdong Province, China, 2021 (N = 1257)

Characteristics	cOR(95% CI)	*P*-value	aOR(95% CI) ‡	*P*-value
Age				
18–22	*ref*	–	*ref*	–
23–28	4.35 (2.56–7.30)	< 0.001*	2.24 (1.24–4.03)	0.007*
29–35	2.13 (1.48–3.09)	< 0.001*	2.14 (1.19–3.87)	0.007*
≥ 36	2.22 (1.54–3.16)	< 0.001*	3.00 (1.72–5.23)	< 0.001*
Marital status				
Unmarried	*ref*	–	*ref*	–
Married	1.13 (0.94–1.87)	0.112	0.93 (0.63–1.39)	0.731
Divorced or separated	2.82 (1.98–4.03)	< 0.001*	1.23 (0.81–1.85)	0.336
Educational level				
Illiterate or elementary	*ref*	–	*ref*	–
Middle school	0.36 (0.26–0.51)	< 0.001*	0.64 (0.44–0.92)	0.015*
High school and above	0.32 (0.21–0.48)	< 0.001*	0.60 (0.37–0.97)	0.037*
Monthly income ($)				
< 3000	*ref*	–	*ref*	–
3000–5000	1.84 (1.14–2.97)	0.053	1.66 (0.99–2.78)	0.053
5000–8000	0.62 (0.36–1.07)	0.084	0.98 (0.54–1.78)	0.943
> 8000	0.30 (0.15–0.59)	0.001*	0.67 (0.32–1.40)	0.284
Workplace				
Middle and low tier	*ref*	–	*ref*	–
High tier	0.15 (0.11–0.21)	< 0.001*	0.21 (0.14–0.30)	< 0.001*
Length of time working in current location				
< 6 months	*ref*	–	*ref*	–
6–12 months	3.70 (2.22–6.17)	< 0.001*	2.64 (1.54–4.54)	< 0.001*
Over 1 year	3.59 (2.24–5.75)	< 0.001*	2.82 (1.71–4.64)	< 0.001*
Have ever occurred a gynecological disease				
No	*ref*	–	*ref*	–
Yes	1.70 (1.23–2.34)	< 0.001*	1.89 (1.35–2.66)	< 0.001*
Have ever received any local health services				
No	*ref*	–	*ref*	–
Yes	0.52 (0.39–0.71)	< 0.001*	0.67 (0.47–0.96)	0.029*
Sexual behavior				
Number of client (past week)				
≤ 3	*ref*	–	*ref*	–
4–6	1.85 (1.11–3.09)	0.019	3.05 (1.73–5.35)	< 0.001*
≥ 7	3.77 (2.39–5.94)	< 0.001*	4.53 (2.74–7.51)	< 0.001*
Consistent condom use with client (past month)				
No	*ref*	–	*ref*	–
Yes	0.11 (0.08–0.16)	< 0.001*	0.16 (0.10–0.23)	< 0.001*
Have ever looked for clients through online software?				
No	*ref*	–	*ref*	–
Yes	6.36 (4.60–8.80)	< 0.001*	4.96 (3.40–7.23)	< 0.001*
Currently the main approach of contraception				
Condom	*ref*	–	*ref*	–
Other contraceptive methods†	1.69 (1.15–2.48)	0.007*	1.77 (1.16–2.70)	0.007*
STIs testing				
Previous chlamydia testing				
No	*ref*	–	*ref*	–
Yes	6.67 (4.92–9.02)	< 0.001*	3.71 (2.67–5.18)	< 0.001*
Previous gonorrhea testing				
No	*ref*	–	*ref*	–
Yes	6.96 (5.14–9.43)	< 0.001*	3.88 (2.77–5.43)	< 0.001*
Gonorrhea testing				
Negative	*ref*	–	*ref*	–
Positive	2.37 (1.08–5.24)	0.032*	1.49 (0.64–3.49)	0.412

^*^P < 0.05

^†^such as oral contraceptive or long-acting contraceptive needle/ring

^‡^ Adjusted for age, workplace, working times in the local area, education level and monthly income were adjusted for each other, all other variables were adjusted for age, workplace, working times in the local area, educational level, and monthly income

### Factors correlated with multiple induced abortion in lifetime

In the multivariable model, multiple abortion was positive with having ever looked for clients through online software (aOR = 4.47, 95% CI 1.80–11.09) after adjusting for age, workplace, working times in the local area, education level, and monthly income (Table 4).

**Table 4:** Factors associated with multiple abortion among FSW who had at least one abortion due to commercial sex in Guangdong Province, China, 2021 (N = 211)

Variables	Total n (%)	Multiple induced abortion		cOR(95%CI)	aOR(95%CI) ‡
		No n (%)	Yes n (%)		
Demographics	211	116 (55.0)	95 (45.0)		
Age					
Mean ± SD	35.13 ± 9.23	35.04 ± 9.21	35.23 ± 9.31		
18–22	18 (8.5)	10 (8.6)	8 (8.4)	*ref*	*ref*
23–28	43 (20.4)	23 (19.8)	20 (21.1)	1.09 (0.36–3.29)	1.20 (0.39–3.75)
29–35	47 (22.3)	28 (24.1)	19 (20.0)	0.85 (0.28–2.54)	0.88 (0.29–2.73)
≥ 36	103 (48.8)	55 (47.5)	48 (50.5)	1.09 (0.40–2.99)	1.08 (0.38–3.09)
Marital status					
Unmarried	64 (30.3)	37 (31.9)	27 (28.4)	*ref*	*ref*
Married	72 (34.1)	46 (39.7)	26 (27.4)	0.78 (0.39–1.55)	0.84 (0.41–1.75)
Divorced	75 (35.6)	33 (28.4)	42 (44.2)	1.74 (0.89–3.42)	1.70 (0.83–3.51)
Educational level					
Illiterate or elementary	80 (37.9)	47 (40.5)	33 (34.8)	*ref*	*ref*
Middle school	95 (45.0)	49 (42.3)	46 (48.4)	1.34 (0.73–2.44)	1.52 (0.80–2.87)
High school and above	36 (17.1)	20 (17.2)	16 (16.8)	1.14 (0.52–2.52)	1.96 (0.78–4.92)
Place of domicile					
Local city	12 (5.7)	5 (4.3)	7 (7.4)	*ref*	*ref*
Other cities in Guangdong province	47 (22.3)	25 (21.6)	22 (23.2)	0.63 (0.17–2.27)	0.66 (0.17–2.59)
Other provinces	152 (72.0)	86 (74.1)	66 (69.4)	0.55 (0.17–1.81)	0.49 (0.14–1.75)
Workplace					
Middle and low tier	167 (79.2)	86 (74.1)	81 (85.3)	*ref*	*ref*
High tier	44 (20.8)	30 (25.9)	14 (14.7)	0.46 (0.25–1.00)	0.52 (0.23–1.16)
Length of time working in current location					
< 6 months	18 (8.5)	11 (9.5)	7 (7.4)	*ref*	*ref*
6–12 months	58 (27.5)	32 (27.6)	26 (27.4)	1.28 (0.43–3.76)	1.52 (0.48–4.81)
Over 1 year	135 (64.0)	73 (62.9)	62 (65.2)	1.34 (0.49–3.65)	1.66 (0.57–4.82)
Have ever occurred a gynecological disease					
No	60 (28.4)	33 (28.5)	27 (28.4)	*ref*	*ref*
Yes	151 (71.6)	83 (71.5)	68 (71.6)	2.82 (1.42–5.60)*	4.47 (1.80–11.09)*
Have ever received any local health services					
No	62 (29.4)	29 (25.0)	33 (34.7)	*ref*	*ref*
Yes	149 (70.6)	87 (75.0)	62 (65.3)	1.60 (0.88–2.90)	1.48 (0.80–2.73)
Sexual behavior					
Number of clients (past week)					
Mean ± SD	9.30 ± 4.56	9.34 ± 4.88	9.25 ± 4.17		
≤ 3	20 (9.5)	13 (11.2)	7 (7.4)	*ref*	*ref*
4–6	45 (21.3)	25 (21.6)	20 (21.0)	1.49 (0.50–4.42)	1.63 (0.51–5.20)
≥ 7	146 (69.2)	78 (67.2)	68 (71.6)	1.62 (0.61–4.29)	1.96 (0.68–5.64)
Consistent condom use with clients (past month)					
No	176 (83.4)	95 (81.9)	81(85.3)	*ref*	*ref*
Yes	35 (16.6)	21 (18.1)	14(14.7)	0.78(0.37–1.64)	1.10(0.49–2.45)
Have ever looked for clients through online software?					
No	52 (24.6)	38 (32.8)	14 (14.7)	*ref*	*ref*
Yes	159(75.4)	78 (67.2)	81 (85.3)	2.82 (1.42–5.60)*	4.47 (1.80–11.09)*
Currently the main approach of contraception					
Condom	175 (82.9)	98 (84.5)	77 (81.1)	*ref*	*ref*
Other contraceptive methods†	36 (17.1)	18 (15.5)	18 (18.9)	1.27 (0.62–2.61)	1.56 (0.71–3.40)
Previous chlamydia testing					
No	88 (41.7)	57 (49.1)	31 (32.6)	*ref*	*ref*
Yes	123 (58.3)	59 (50.9)	64 (67.4)	1.99 (1.14–3.50)*	1.75 (0.95–3.24)
Previous gonorrhea testing					
No	89 (42.2)	57 (49.1)	32 2(33.7)	*ref*	*ref*
Yes	122 (57.8)	59 (50.9)	63(66.3)	1.90(1.09–3.33)*	1.72 (0.94–3.15)
Syphilis testing					
Negative	205 (97.16)	114 (98.28)	91 (95.79)	*ref*	*ref*
Positive	6 (2.84)	2 (1.72)	4 (4.21)	2.51 (0.45–13.97)	0.54 (0.09–3.18)
Chlamydia testing					
Negative	198 (93.8)	109 (94.0)	89 (93.7)	*ref*	*ref*
Positive	13 (6.2)	7 (6.0)	6 (6.3)	1.05 (0.34–3.24)	0.96 (0.30–3.08)
Gonorrhea testing					
Negative	205 (97.16)	112 (96.55)	93 (97.9)	*ref*	*ref*
Positive	6 (2.84)	4 (3.45)	2 (2.1)	0.60 (0.11–3.36)	1.11 (0.18–6.76)
Combined STIs testing					
Negative	189 (89.6)	105 (90.5)	84 (88.4)	*ref*	*ref*
Positive	22 (10.4)	11 (9.5)	11 (11.6)	1.25 (0.52–3.03)	0.78 (0.31–1.98)

^*^ P < 0.05

^†^such as oral contraceptive or long-acting contraceptive needle/ring

^‡^Adjusted for age, workplace, working times in the local area, education level and monthly income were adjusted for each other, all other variables were adjusted for age, workplace, working times in the local area, educational level, and monthly income

## Discussion

Our study indicates that FSW in South China are at high risk of unintended pregnancies and induced abortions. To our knowledge, this is the first study to investigate unintended pregnancy, and its associated factors among FSW in south China. Findings from this study could provide insights for the development of policies aimed at reducing unintended pregnancies, improving abortion care, as well as enhancing family planning programs targeted at FSW.

Unintended pregnancy due to commercial sex is prevalent among FSW in South China, with a similar rate observed in other low- and middle- countries such as Benin (16.4%) [9], Rwanda (26.3%) [20], and the Caribbean region (22.5%) [21]. This high rate can be attributed to the following reasons. Primarily, FSW often encountered financial burdens and difficulties in condom use negotiation [22, 23], leading to accepting more clients and engaging in more unprotected sex. Secondly, the majority of FSW are unstable and transient due to the criminalization of sex work in China [24], rendering it challenging for existing interventions to reach and assist them. Furthermore, family planning is not given the same priority in the current strategies for FSW health as it is in HIV/STIs prevention [25], resulting in a dearth of knowledge and utilization of relevant health services. Given the high prevalence of unintended pregnancies and the marginalized nature of FSW, exploring effective strategies to improve the knowledge and utilization of family planning service for them is needed. The Joint United Nations Programme on HIV/AIDS (UNAIDS) recommends prioritizing linkages between family planning and HIV/ STIs to help curb the HIV and pregnancy epidemic [26]. Several low- and middle-income countries have implemented this recommendation and it has been demonstrated to be cost-effective in reducing the prevalence of unintended pregnancy among FSW [27, 28]. Therefore, it is vital to integrate family planning service into current HIV/ STIs programs to mitigate the prevalence of unintended pregnancies for Chinese FSW.

Our findings indicate that the percentage of FSW who experienced unintended pregnancy that ended in induced abortion is remarkably high (96.7%), surpassing the percentage in Benin (67.6%) [9] and Tete (35.9%) [1]. The high percentage of induced abortion may be attributed to the fear of losing clients and income, the violence from regular partners or family [8], the accusation of traditional social culture [29] and the legality of abortion compared to other countries [30]. A systematic review has revealed that abortion is the leading cause of mortality among FSW, as they often experience more health complications from abortion [10], especially with multiple abortion. In our present study, we found that nearly half of FSW reported having undergone more than one lifetime abortion. This discovery emphasizes the need to decrease the incidence of repeat abortion to shield them from the adverse effects of such procedures. The World Health Organization has advocated for the provision of abortion care for FSW [31]. Researches have demonstrated that offering post-abortion care to patients before their discharge from medical facilities can effectively reduce the recurrence of abortion [32, 33]. Moreover, many countries such as Zimbabwe [34] and Uganda [35], have recommended the expansion of knowledge and accessibility of post-abortion care for FSW. Nonetheless, less than half of females in China avail themselves of post-abortion care, and there is limited information about the utilization of such care among Chinese FSW. Hence, it is imperative for public healthcare institutions to establish and promote anonymous abortion services, ensuring that FSW can access safe and confidential services. Concurrently, healthcare facilities, alongside communities, should facilitate comprehensive post-abortion care and promote abortion education to FSW in China.

We found that few FSW (12.6%) have used modern non-barrier contraception. Despite the reliance on condoms as the primary form of pregnancy control for FSW, the risk of unintended pregnancies persists due to inconsistent condom use and condom failure [36]. Non-barrier contraceptive methods, especially long-acting reversible contraceptives, emerge as a dependable alternative, significantly reducing the likelihood of unintended pregnancies among FSW [37]. However, the uptake of non-barrier contraception among FSW in our study is lower than rates observed in similar populations in Cameroon [4] and Russia [38]. This discrepancy can be attributed to educational programs that predominantly promote condom use, neglecting to inform FSW about other modern non-barrier contraception [6]. Additionally, a considerable segment of unmarried or divorced FSW fail to access complimentary contraceptive services, including long-acting reversible contraceptives, despite China's policy of providing non-barrier contraceptives at no cost to women. This policy is depended on marital status verification and is restricted to select venues, such as primary healthcare centers [24]. The necessity for additional intervention is underscored by these reasons, advocating for improved knowledge and access to non-barrier contraception for FSW. More innovative and comprehensive strategies are being implemented in many countries to disseminate knowledge and broaden the availability of non-barrier contraception, incorporating peer-driven [39] and mobile-based [40] interventions. Moreover, WHO endorses dual protection as vital for preventing both unintended pregnancies and HIV, reinforcing the need for dual protection—combining condoms with non-barrier contraception—to prevent both unintended pregnancies and reduce HIV/ STIs transmission [41]. Given the high burden of STIs and unintended pregnancies among FSW, it is essential to add modern non-barrier contraception and dual protection in current contraceptive promotion programs for HIV/STIs prevention among Chinese FSW.

### Strengths and limitations of this study

This study is the first to examine the correlates of unintended pregnancy due to commercial sex among FSW in South China, providing valuable insights for interventions aimed at reducing unintended pregnancy in this population. However, the present study has several limitations. First, the cross-sectional study design restricts our ability to ascertain the temporal direction of the observed associations. Second, data on unintended pregnancies and abortion came from self-reports by FSW participating in the study, which can lead to information bias. Third, because we collected data on lifetime abortion, the study could not distinguish whether multiple abortion was the result of unintended pregnancies due to commercial sex, limiting our ability to assess the direct association between unintended pregnancies and multiple abortion. Finally, even though we have a large sample size, the recruitment of FSW in this study was non-random and limited to cities with extensive experience of outreach services, potentially limiting the generalizability of the findings to other cities.

## Conclusion

In conclusion, our study underscores the high prevalence of unintended pregnancies and induced abortion among FSW in South China. Our findings emphasize the importance of integrating family planning services within existing HIV/STIs programs to meet FSW’s needs of reproductive health and improving the service of post-abortion care to reduce the hazard resulting from induced abortion. It also advocates for strategies that incorporate non-barrier contraceptive methods into current health promotion and services, thereby broadening the contraceptive choices available to this population.

## Data Availability

The dataset used in the study are available from the corresponding author on reasonable request.
